# Deep Learning-Driven Estimation of Centiloid Scales from Amyloid PET Images with ^11^C-PiB and ^18^F-Labeled Tracers in Alzheimer’s Disease

**DOI:** 10.3390/brainsci14040406

**Published:** 2024-04-21

**Authors:** Tensho Yamao, Kenta Miwa, Yuta Kaneko, Noriyuki Takahashi, Noriaki Miyaji, Koki Hasegawa, Kei Wagatsuma, Yuto Kamitaka, Hiroshi Ito, Hiroshi Matsuda

**Affiliations:** 1 Department of Radiological Sciences, School of Health Science, Fukushima Medical University, Fukushima 960-8516, Japan; t.yamao0522@gmail.com (T.Y.);; 2 Department of Radiology, Fukushima Medical University Hospital, Fukushima 960-1295, Japan; 3 School of Allied Health Sciences, Kitasato University, Tokyo 252-0373, Japan; 4 Research Team for Neuroimaging, Tokyo Metropolitan Institute for Geriatrics and Gerontology, Tokyo 173-0015, Japan; 5 Department of Radiology and Nuclear Medicine, Fukushima Medical University, Fukushima 960-1295, Japan; 6 Department of Biofunctional Imaging, Fukushima Medical University, Fukushima 960-1295, Japan

**Keywords:** amyloid PET, deep learning, centiloid scale

## Abstract

Background: Standard methods for deriving Centiloid scales from amyloid PET images are time-consuming and require considerable expert knowledge. We aimed to develop a deep learning method of automating Centiloid scale calculations from amyloid PET images with ^11^C-Pittsburgh Compound-B (PiB) tracer and assess its applicability to ^18^F-labeled tracers without retraining. Methods: We trained models on 231 ^11^C-PiB amyloid PET images using a 50-layer 3D ResNet architecture. The models predicted the Centiloid scale, and accuracy was assessed using mean absolute error (MAE), linear regression analysis, and Bland–Altman plots. Results: The MAEs for Alzheimer’s disease (AD) and young controls (YC) were 8.54 and 2.61, respectively, using ^11^C-PiB, and 8.66 and 3.56, respectively, using ^18^F-NAV4694. The MAEs for AD and YC were higher with ^18^F-florbetaben (39.8 and 7.13, respectively) and ^18^F-florbetapir (40.5 and 12.4, respectively), and the error rate was moderate for ^18^F-flutemetamol (21.3 and 4.03, respectively). Linear regression yielded a slope of 1.00, intercept of 1.26, and R^2^ of 0.956, with a mean bias of −1.31 in the Centiloid scale prediction. Conclusions: We propose a deep learning means of directly predicting the Centiloid scale from amyloid PET images in a native space. Transferring the model trained on ^11^C-PiB directly to ^18^F-NAV4694 without retraining was feasible.

## 1. Introduction

Alzheimer’s disease (AD) is a major cause of dementia characterized by amyloid-β plaques, hyperphosphorylated tau protein, and brain atrophy [1,2]. Amyloid-β, a key hallmark of AD, begins to accumulate over two decades before the onset of symptoms. Non-invasive amyloid positron emission tomography (PET) allows the early detection of amyloid-β accumulation, which is critical for a differential diagnosis of AD. Fluorine-18-labeled amyloid tracers, such as ^18^F-florbetaben, ^18^F-flutemetamol, and ^18^F-florbetapir, are currently available for routine clinical amyloid PET imaging. On the other hand, ^11^C-PiB and ^18^F-NAV4694 are available for research only. These amyloid PET tracers visualize the distribution of amyloid-β despite different chemical architectures. Confirmation of amyloid pathology by amyloid PET or cerebrospinal fluid (CSF) tests is essential for the timely administration of disease-modifying drugs [3].

While amyloid PET results are often visually assessed as negative or positive in clinical practice, quantitative evaluations are essential for clinical investigations and the development of drugs to treat AD. The standardized uptake value ratio (SUVR) is a common quantitative measure, but it varies depending on the region of interest and the tracer. The Global Alzheimer’s Association Interactive Network (GAAIN) introduced the Centiloid scale to address these inconsistencies [4]. The Centiloid scale is defined by linearly scaling the average SUVR value to 0 for subjects with a high certainty of being amyloid-negative and to 100 for typical AD patients. The reproducibility of the Centiloid scale calculation can be verified by quality control using available PET and MRI datasets on the GAAIN website. Although the Centiloid scale is defined based on ^11^C-PiB data, conversion of the SUVR of ^18^F-labeled amyloid tracers to the ^11^C-PiB equivalent of the Centiloid scale allows the direct comparison of quantitative values between different tracers. However, the calculation of the Centiloid scale requires several conditions. First, a three-dimensional T1-weighted image covering the region from the vertex to the whole cerebellum is required from the same subject. Then, manual image analysis is required to calculate the Centiloid scale. It involves co-registration of the PET and MRI images of the same subject, anatomical standardization, VOI analysis using specific VOIs, and the conversion process from SUVR to the Centiloid scale. To overcome this, a simple method of calculating the Centiloid scale using low-dose CT instead of MRI has been reported [5,6,7]. However, manual image analysis and acquisition of anatomical images are still required, and no studies have been reported that automatically calculate the Centiloid scale using only PET images. Quantitative analysis using only PET images allows the evaluation of subjects without the corresponding MRI data.

Recently, deep learning methods in amyloid PET have shown exceptional performance in areas such as classification [8], visual interpretation support [9], the prediction of cognitive decline [10], and image restoration [11]. In addition, deep learning has been applied to predict quantitative values from amyloid PET images. Deep learning-based anatomical standardization method for ^18^F-florbetaben or ^18^F-flutemetamol PET without MRI has been proposed [12]. A deep learning model was developed to quantify SUVR from ^18^F-florbetapir or ^18^F-florbetaben PET images in a native space [13]. A deep learning quantification of the SUVR of an ^18^F-florbetapir PET image using a pretrained 2D CNN has also been reported [14]. The use of generative adversarial network model to generate structural MRI image from ^18^F-florbetapir PET image has been proposed for the quantification of PET alone [15]. However, most of these techniques still rely on PET and MRI preprocessing. In particular, the Centiloid scale has not been directly predicted using deep learning. Since the Centiloid scale is converted from the SUVR calculated with the specific volume of interest (VOI), a deep learning model for the Centiloid scale appears to be of importance. The advantages of 3D convolutional neural network (CNN), which can account for continuity between slices, are significant for an accurate Centiloid scale prediction. The direct comparison of the different tracers is also an important part of the Centiloid scale. Therefore, it is necessary to evaluate the model using a number of amyloid tracers, not just a single one.

In this study, we aimed to fill this gap by directly predicting the Centiloid scale from amyloid PET images without MRI using a 3D CNN. In addition, we investigated whether a 3D CNN constructed with ^11^C-PiB could be applied to ^18^F-NAV4694, ^18^F-florbetaben, ^18^F-flutemetamol, and ^18^F-florbetapir without retraining. These PET tracers have the common feature of binding to amyloid-β deposition, suggesting the potential applicability of the deep learning model to different amyloid tracers. These PET data are available from GAAIN and conversion methods to the ^11^C-PiB-equivalent Centiloid scale have been reported. The ability to apply various amyloid tracers enhances the utility of the Centiloid scale calculation methods, which is a significant advantage in their adoption for research and clinical use.

## 2. Materials and Methods

### 2.1. Dataset

We downloaded 79 amyloid ^11^C-PiB [4] and 210 amyloid ^18^F-NAV4694 [16], ^18^F-Florbetaben [17], ^18^F-Flutemetamol [18], and ^18^F-Florbetapir [19] PET images from the GAAIN database (https://www.gaain.org/centiloid-project accessed on 6 October 2022). Table 1 shows details of the amyloid PET dataset. The ground truth of the Centiloid scale value for deep learning prediction is published on the GAAIN website. A Centiloid scale value calibrated to ^11^C-PiB is provided for ^18^F-labeled amyloid PET. We employed these PET datasets to construct a predictive model for the Centiloid scale and to evaluate its applicability across multiple tracers. In the dataset of ^18^F-labeled amyloid PET, ^11^C-PiB PET imaging was also conducted on same subjects. Therefore, we utilized a total of 289 amyloid ^11^C PiB images from different repositories. ^11^C-PiB PET scans were performed on 34 healthy subjects and 45 patients with AD, for a total of 79 participants [4,20,21,22,23,24,25,26]. ^11^C-PiB PET image was acquired 50 to 70 min after injection. The acquisition time for ^11^C-PiB was consistent across all datasets. The PET scanners used a BioGraph TruePoint TrueV (Siemens Healthineers, Erlangen, Germany), an ECAT Exact HR+ (Siemens), an ECAT Exact HR (Siemens), and an Allegro PET camera (Philips Medical Systems, Eindhoven, The Netherlands). Images were reconstructed using the filtered back projection (FBP), the 3D row-action maximum likelihood algorithm (RAMLA), and the ordered subsets expectation maximization (OSEM). ^11^C-PiB and ^18^F-NAV4694 PET scans were performed on 10 young controls, 25 elderly controls, 10 patients with mild cognitive impairment (MCI), 7 patients with mild AD, and 3 patients with frontotemporal dementia (FTD). The ^18^F-NAV4694 PET image was acquired 50 to 70 min after injection using an Allegro PET camera in the 3D mode. Images were reconstructed using the 3D RAMLA [16]. ^11^C-PiB and ^18^F-florbetaben PET images were performed on 10 young controls, 6 elderly controls, 10 patients with MCI, 7 patients with AD, and 3 patients with FTD. ^18^F-florbetaben PET image was acquired 90 to 110 min after injection using an Allegro PET camera [27]. Images were reconstructed using the 3D RAMLA and the line of response (LOR) RAMLA. ^11^C-PiB and ^18^F-flutemetamol PET images were performed on 24 young controls, 10 elderly controls, 20 patients with MCI, and 20 patients with AD. ^18^F-flutemetamol PET images were acquired 90 to 110 min after injection using a 16-slice Biograph (Siemens), an ECAT EXACT HR+, a GE Advance scanner (GE Healthcare, Milwaukee, WI, USA), a Discovery RX, and a Discovery RXT (GE Healthcare) [18,28,29]. Images were reconstructed by the FBP and the OSEM. ^18^F-florbetapir PET image was obtained for 13 young controls, 6 elderly controls, 3 at-risk elderly controls, 7 patients with MCI, 3 patients with possible AD, and 14 patients with AD. ^18^F-florbetapir PET image was acquired 50 to 60 min after injection using an ECAT Exact HR+ in the 2D mode with 2D-OSEM, a Gemini TF 64 (Philips) in the 3D mode with LOR-RAMLA, and a GE Advance scanner in the 2D mode with Fourier rebinning iterative reconstruction algorithm [19].

### 2.2. Deep Learning Model Architecture for Predicting Centiloid Scale

**Figure 1:**
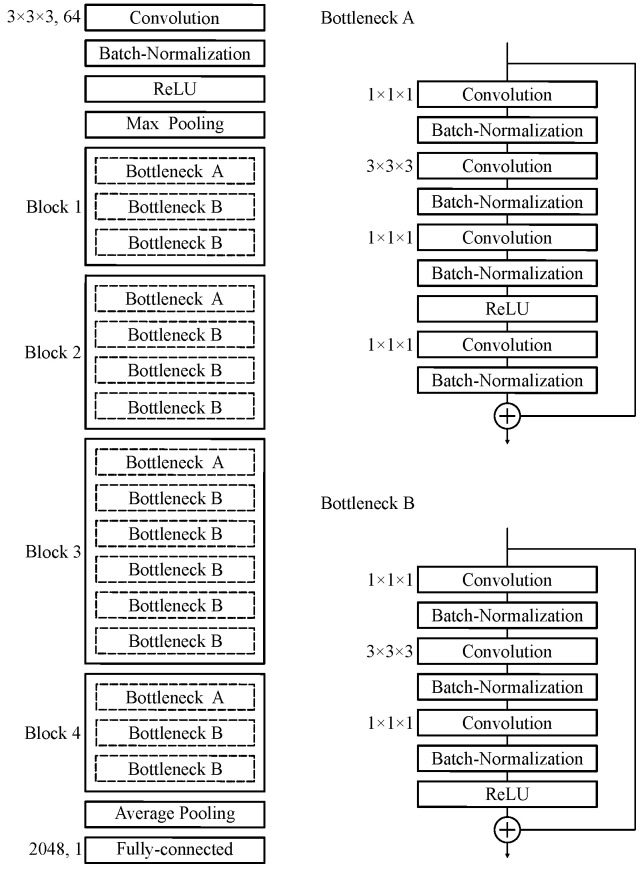
Architecture of 50-layer 3D ResNet for predicting Centiloid scale from native amyloid PET images. The width of the convolutional kernel was set to 1 × 1 × 1 or 3 × 3 × 3.

The Centiloid scale was predicted from amyloid PET images using the 50-layer three-dimensional (3D) ResNet architecture (https://github.com/xmuyzz/3D-CNN-PyTorch accessed on 6 December 2023). This deep learning model was modified from the standard 3D ResNet to address the specific complexities of processing PET images for AD. ResNet facilitates effective learning in the deepest models through skip connection. In addition, the ability of 3D CNN to process volumetric data makes it particularly useful for PET data analysis in AD, where it can effectively capture the spatial complexity of local amyloid deposition. It features a comprehensive design that includes 3D convolutional layers for spatial data processing, batch normalization to accelerate training and improve model performance, and rectified linear units (ReLUs) for nonlinear transformations. The architecture also includes a max-pooling layer to reduce dimensionality and improve feature extraction, followed by four sequential layer blocks carefully constructed with bottleneck blocks. These blocks are designed to deepen the network without increasing its complexity or computational load through a combination of 3D convolution, batch normalization, ReLUs, and down sampling layers. This allows the model to learn more complex features from the PET images with greater efficiency. Sequential layer blocks consisting of 3, 4, 6, and 3 bottleneck blocks allow the model to adaptively refine its predictions, making it highly effective for medical imaging tasks. An average-pooling layer follows these blocks, leading to a fully connected layer that culminates the network architecture and facilitates the final prediction of the Centiloid scale. Figure 1 shows the structure of our deep learning model. A network model was implemented on the following system environment: Intel(R) Xenon Gold 5222 3.80 GHz; NVIDIA RTX A6000 video card with 24 GB of video memory; CUDA v. 11.6; PyTorch v. 1.12.1; Python v. 3.8.13; and Ubuntu 18.04 LTS.

### 2.3. Deep Learning Training and Test Phase

**Figure 2:**
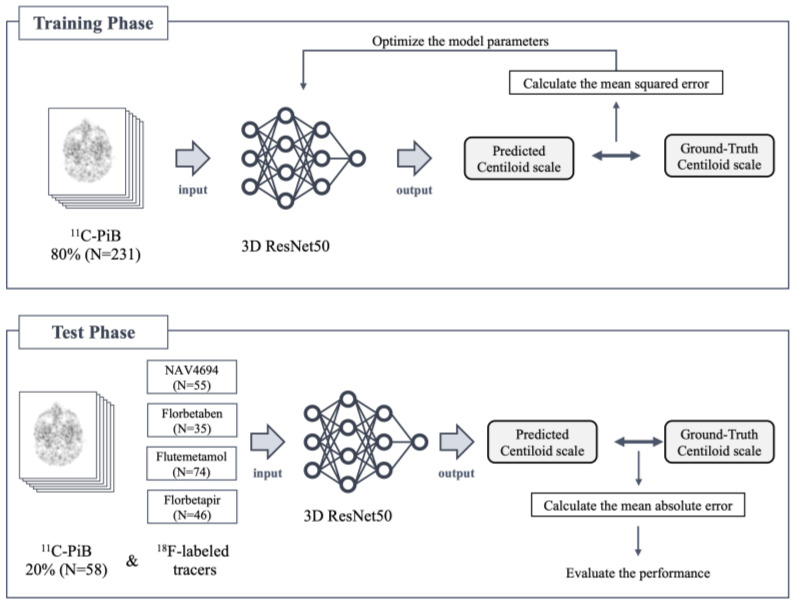
Scheme of deep learning training and testing phases.

Figure 2 shows the comprehensive training and testing processes of our deep learning analysis. The deep learning model was trained on 231 (80%) of the 289 available ^11^C-PiB amyloid PET images and tested on 58 (20%) of the 289 images, with ^18^F-amyloid PET images included in the testing phase without further training. It has been demonstrated that deep learning models trained on ^18^F-florbetapir PET images can be accurately applied to ^18^F-florbetaben images without the need for retraining [13]. This study extends this approach by applying a model trained on ^11^C-PiB to four types of ^18^F-labeled amyloid tracers.

All pixel values of less than 0 were adjusted to 0 because PET images reconstructed using the FBP algorithm were included. The training images were randomly rotated (between −10° and 10°) and scaled to increase the robustness of the model. Since the original matrix size were not uniform, the images were resized to 128 × 128 × 128 voxels. In order to ensure uniformity across the dataset and enhance the model’s ability to learn from the PET images effectively, the voxel intensity was normalized using min–max normalization for model input.

The mean square error (MSE) was used for the loss function and adaptive moment (Adam) estimation of the optimization algorithm.

$$MSE=\frac{1}{n}\sum_{i=1}^{n}{\left(y_{i}-{\hat{y}}_{i}\right)}^{2}$$

where *n* is the number of PET images, *y_i_* is the ground truth Centiloid scale, and ${\hat{y}}_{i}$ is the Centiloid scale computed by our deep learning model. The use of the MSE as the loss function is advantageous because it emphasizes larger errors by squaring the error values, thus causing the model to focus more on reducing these errors during training. The learning rate was 0.0001, and the batch size was 4. To avoid overfitting, the training phase was terminated when performance did not improve over 20 consecutive epochs. Thus, the number of epochs was 73.

The ability to predict the Centiloid scale was evaluated using the mean absolute error (MAE) on the test dataset.

$$MAE=\frac{1}{n}\sum_{i=1}^{n}\left|y_{i}-{\hat{y}}_{i}\right|$$

where *n* is the number of PET images, *y_i_* is the ground truth Centiloid scale, and ${\hat{y}}_{i}$ is the Centiloid scale computed by our deep learning model. Using the MAE as a performance metric has the advantage of providing a direct interpretation of the average prediction error in the same units as the predicted value.

### 2.4. Statistical Analysis

The difference in predictive performance between ADs and YCs was tested using the Mann–Whitney U test with a significance level of 0.05. One-way analysis of variance (ANOVA) was used to evaluate differences in predictive performance between different tracers. In cases where one-way ANOVA indicated significant differences, Bonferroni-corrected post hoc tests were used to identify specific groups. The correlation between the deep learning approach and the ground truth was assessed using Pearson correlation analysis. The slope, intercept, and coefficient of determination were obtained using linear regression analyses of the ground truth and predicted values of the test set. Centiloid scale concordance between deep learning prediction and ground truth was assessed using Bland–Altman plots. All data were statistically analyzed using Python v. 3.8.13, scikit-learn v. 1.1.2, and SciPy v. 1.8.13.

## 3. Results

**Figure 3:**
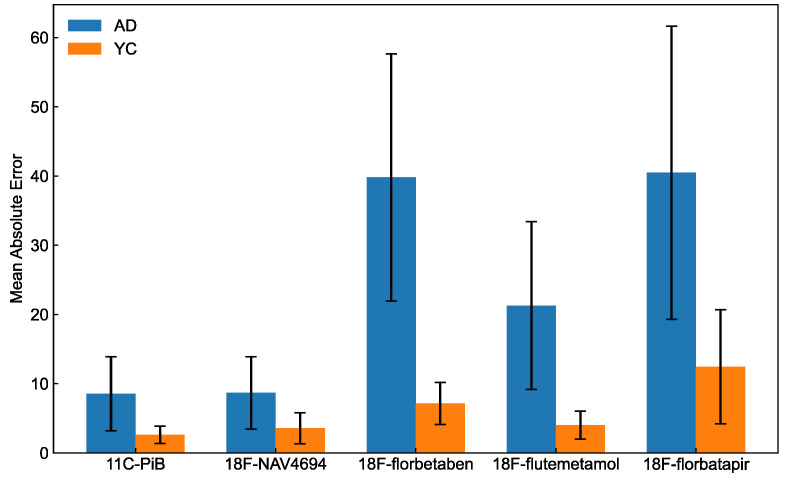
Performance of deep learning ability to predict the Centiloid scale.

The evaluation of the predictive accuracy of deep learning was carefully performed using the designated test set. Figure 3 shows a detailed comparison of the MAE for both ^11^C-PiB and various ^18^F-labeled amyloid tracers, delineating the results for young controls (YCs) and AD patients. The MAEs for AD and YC were 8.54 and 2.61, respectively, using ^11^C-PiB, and 8.66 and 3.56, respectively, using ^18^F-NAV4694. The MAEs for AD and YC were higher with ^18^F-florbetaben (39.8 and 7.13, respectively) and ^18^F-florbetapir (40.5 and 12.4, respectively), and the error rate was moderate for ^18^F-flutemetamol (21.3 and 4.03, respectively). AD patients had higher MAE values for all tracers compared to YC, indicating a divergence in predictive accuracy between these groups. The MAE was significantly higher for AD than YC for all tracers (^11^C-PiB, U = 570.0 and *p* < 0.001; ^18^F-NAV4694, U = 319.0 and *p* < 0.05; ^18^F-florbetaben, U = 217.0 and *p* < 0.001; ^18^F-flutemetamol, U = 958.0 and *p* < 0.001; ^18^F-florbetapir, U = 342.0 and *p* < 0.05;). Significant differences between groups were found for the amyloid PET tracers by one-way ANOVA (F = 17.8 and *p* < 0.001). Significant differences between groups were found for the amyloid PET tracers by one-way ANOVA (F = 17.8 and *p* < 0.001). Post hoc tests confirmed a significant difference in the following six groups: ^11^C-PiB and ^18^F-florbetaben (*p* < 0.001), ^11^C-PiB and ^18^F-florbetapir (*p* < 0. 001), ^18^F-NAV4694 and ^18^F-florbetaben (*p* < 0.001), ^18^F-NAV4694 and ^18^F-florbetapir (*p* < 0.001), ^18^F-florbetaben and ^18^F-flutemetamol (*p* < 0.001), and ^18^F-florbetapir and ^18^F-flutemetamol (*p* < 0.001).

**Figure 4:**
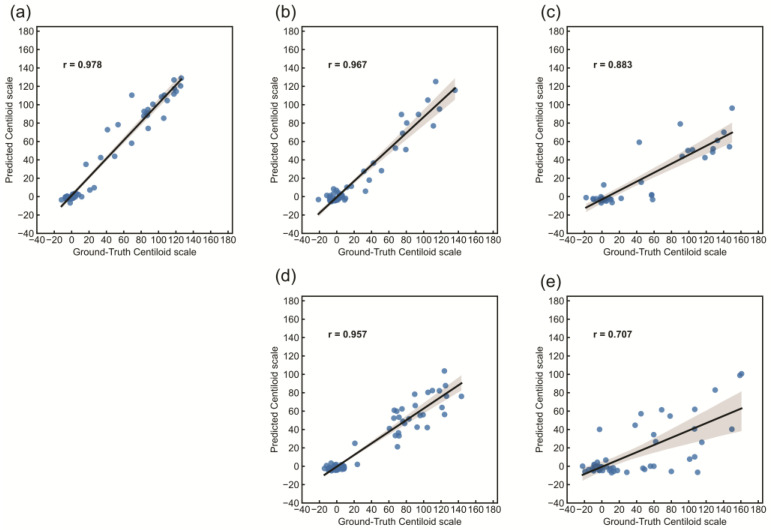
Scatter plot between the ground truth and the predicted Centiloid scale. The results of the linear regression analysis are shown with a black line, and r is the correlation coefficient: (**a**) ^11^C-PiB, (**b**) ^18^F-NAV4694, (**c**) ^18^F-florbetaben, (**d**) ^18^F-flutemetamol, and (**e**) ^18^F-florbetapir.

Figure 4 shows scatter plots of Pearson correlation analyses. The correlation between deep learning prediction and the ground truth of the Centiloid scale was significant (r = 0.978; *p* < 0.001). Linear regression analysis yielded the following values: slope, 1.00; intercept, 1.26; and coefficient of determination, 0.956. The Centiloid scale calculated by our deep learning model was equivalent to that of the GAAIN Centiloid Project (slope, 0.98–1.02; intercept, −2–2; R^2^ correlation coefficient > 0.98). The prediction accuracy of 11C-PiB (r = 0.978), ^18^F-NAV4694 (r = 0.967), and ^18^F-flutemetamol (r = 0.957) without retraining was comparable. The correlation between the ground truth and the predicted Centiloid scale was lower for ^18^F-florbetaben (r = 0.883) and ^18^F-florbetapir (r = 0.707) than for the other tracers. 

**Figure 5:**
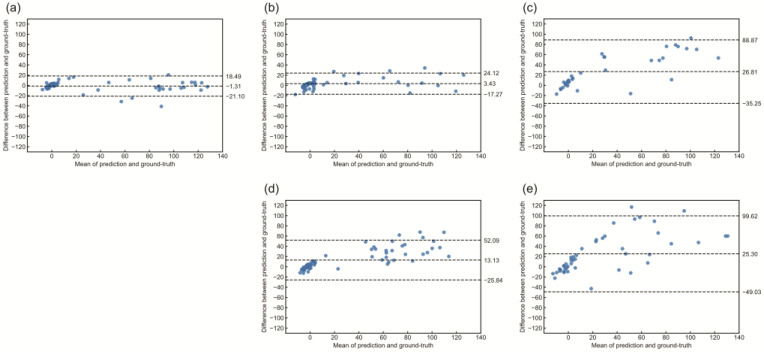
Bland-Altman plot comparing the ground truth and the predicted Centiloid scale. Blue circles represent an individual measurement. The middle-dashed line represents the mean difference, while the upper and lower dashed lines indicate the limits of agreement (mean difference ± 1.96 standard deviations): (**a**) ^11^C-PiB, (**b**) ^18^F-NAV4694, (**c**) ^18^F-florbetaben, (**d**) ^18^F-flutemetamol, and (**e**) ^18^F-florbetapir.

Figure 5 shows Bland–Altman plots in which the mean bias of the Centiloid scale between deep learning prediction and ground truth was −1.31, with 95% acceptable limits of −21.10 and 18.49.

## 4. Discussion

Our team has developed a deep learning system specifically designed to predict the Centiloid scale from amyloid PET images using an advanced 50-layer 3D ResNet architecture. Since the conventional calculation of the Centiloid Scale requires MR images and complex quantitative analysis, our deep learning-based approach offers significant clinical advantages and streamlines the assessment process. To our knowledge, this is the first application of deep learning to derive the Centiloid scale directly from ^11^C-PiB amyloid PET images in their native space, marking a significant milestone in neuroimaging analysis. In addition, we investigated the feasibility of applying models trained on ^11^C-PiB data to ^18^F-labeled amyloid tracers without the need for further training. Our exploration extended to evaluating the model’s performance across a spectrum of individuals with normal cognitive function to those diagnosed with Alzheimer’s disease, with the goal of demonstrating the versatility and potential of our deep learning system to improve diagnostic processes for neurodegenerative diseases.

The Centiloid scale was directly and accurately computed from ^11^C-PiB amyloid PET images in a native space using deep learning (Figure 3). The prediction accuracy was significantly higher in the YC than the AD group (*p* < 0.05). The reported cut-off for the Centiloid scale for normal cognition and AD is 10–35 [6,30,31,32,33]. The present findings showed that the respective Centiloid ranges for YC and AD were −18.26–28.7 and −22.2–160.7. The AD group included patients with frontotemporal dementia (FTD) and mild cognitive impairment (MCI) who had to recognize various feature patterns from images. Thus, we assumed that such patients would have a higher learning difficulty than cognitively typical people.

**Figure 6:**
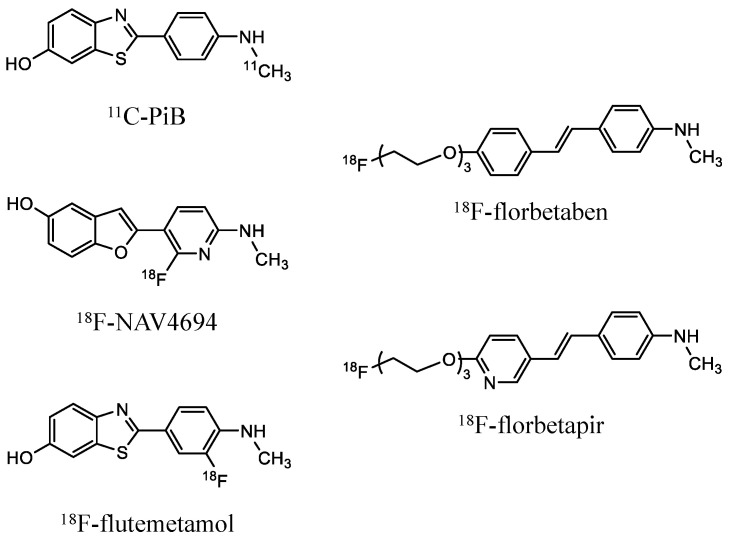
Chemical architecture of amyloid imaging tracers.

The prediction performance of the Centiloid scale using deep learning differed among the amyloid tracers (Figure 4 and Figure 5). This might have been due to the structure or dynamic range of each tracer (Figure 6). The deep learning prediction for ^18^F-NAV4694 was almost identical to that of ^11^C-PiB. Time–activity curves and blood clearance of ^18^F-NAV4694 and ^11^C-PiB are similar [34] and have the same dynamic range [16,35]. Furthermore, ^18^F-NAV4694 has higher specific and lower non-specific accumulation than other ^18^F-labeled amyloid tracers [36,37,38]. The predictive performance of ^18^F-flutemetamol was the same as that of PiB for YC, but the error for AD was high. The slightly wider dynamic range of ^18^F-flutemetamol compared with ^18^F-florbetaben and ^18^F-florbetapir [39,40] resulted in better prediction accuracy. High non-specific binding in white matter might affect prediction accuracy. Errors were the highest for ^18^F-florbetapir and ^18^F-florbetaben. A quantitative value prediction model trained with ^18^F-florbetapir can be applied to ^18^F-florbetaben without retraining [13]. The structures of the thioflavin derivatives ^11^C-PiB, ^18F^-NAV4694, and ^18^F-flutemetamol are similar [41]. On the other hand, ^18^F-florbetapir and ^18^F-florbetaben are stilbene derivatives of Congo red [41]. Their distribution in the brain varies due to these structural differences. The model that learned with ^11^C-PiB had high predictive performance with ^18^F-NAV4694 and ^18^F-flutemetamol. In contrast, the model that learned with ^18^F-florbetapir had high predictive performance with ^18^F-florbetaben. Therefore, the translucency of the model might differ depending on the chemical structure and the distribution of each drug in the brain even when the amyloid PET tracer is the same. When building deep learning models for multiple tracers, chemical structure and dynamic range have a significant impact on model performance. Therefore, not only the deep learning model but also the knowledge of the tracers become critical in model development and application. When using a model with tracers other than those used in training, it is important to be careful about quantitative accuracy. It has been shown that the higher the structural or imagistic similarity between the tracers, the higher the applicability of the deep learning model. This is not limited to amyloid PET imaging but is also expected to apply to other PET imaging modalities, such as tau PET.

The correlation and linearity between the ground truth Centiloid scale and the deep learning predictions show excellent accuracy (Figure 5). Deep learning methods significantly streamline the calculation of the Centiloid scale by eliminating the need for extensive PET and MRI image analysis and, thus, are not affected by variations in image analysis processes such as co-registration and anatomical standardization. In addition, this deep learning-based Centiloid prediction minimizes quantification variability due to reference region selection and efficiently computes the Centiloid scale from native-space amyloid PET images in just 0.10 s. The absence of preprocessing bias in the Centiloid scale computed by deep learning, due to its reliance on native-space PET images, and the elimination of variation due to slice selection, unlike in 2D models, by using a 3D model, highlight the robustness and precision of this innovative approach. A 3D model has a much larger number of parameters than a 2D model. A 2D ResNet50, with an input size of 224 × 224 × 3 pixels, has approximately 2.56 million parameters, while a 3D ResNet50, with an input size of 224 × 224 × 224 pixels, has approximately 48 million parameters. The number of 3D ResNet50 parameters used in this study is consistent with those of the previous studies [42]. Therefore, we consider the structure of our 3D ResNet to be standard in the field.

This study has several limitations. The dataset was relatively small. More data regarding ^18^F-labeled amyloid tracers and amyloid PET-positive individuals are needed from larger samples. The predictive performance can potentially be improved, particularly in the 12–30 Centiloid range. A transparent explanation for the decision making process used by deep learning models is essential. The disadvantage of black-box deep learning is that the underlying decision basis must be determined by visualization using means such as heat maps. The model must be specific to each type of amyloid tracer and carefully selected to avoid inaccurate predictions. In this study, there was a duplication of subjects across different amyloid PET tracers. Subjects who underwent PET imaging with ^18^F-labeled amyloid tracers also underwent imaging with ^11^C-PiB. The ^11^C-PiB PET images were used to train the model, while the ^18^F-labeled images were used to test the model. The possible overestimation of predictive results is due to the duplication of subjects. However, despite the similar distribution patterns of several amyloid tracers, the images are not identical. In fact, differences in predictive performance were observed among the tracers in ^18^F-labeled amyloid PET, and no overestimation by the model was found. In order to improve predictive performance, ensuring a sufficient amount of data for model training was considered critical. In future research, the use of larger datasets could improve the prediction accuracy of the model. Subjects with different cognitive status and multiple amyloid PET tracers must be included.

## 5. Conclusions

We developed a deep learning method with 3D CNN to predict the Centiloid scale from amyloid PET images without MRI images. In addition, the applicability of a 3D CNN constructed with ^11^C-PiB to ^18^F-NAV4694, ^18^F-florbetaben, ^18^F-flutemetamol, and ^18^F-florbetapyr without retraining was investigated. Our method eliminates manual image analysis and provides consistent, reproducible quantitative results. The advanced redirection of deep learning models for tracers with similar properties was feasible. The current findings may not be limited to amyloid PET but may be applicable to the deep learning approach for any PET imaging.

## Figures and Tables

**Table 1:** Clinical demographics of GAAIN dataset for amyloid PET. ^18^F-labeled and ^11^C-PiB amyloid PET images were acquired in one subject each.

PET Tracer	Total	Controls	Patients
^11^C-PiB	79	34	45
^18^F-NAV4694 and ^11^C-PiB	55	10	45
^18^F-Florbetaben and ^11^C-PiB	35	10	25
^18^F-Flutemetamol and ^11^C-PiB	74	24	50
^18^F-Florbetapir and ^11^C-PiB	46	13	33

## Data Availability

The datasets used and/or analyzed during the current study are available from the corresponding author upon reasonable request. The data are not publicly available due to copyright policy of the institutions.
